# An Evaluation of Health Impact Assessments in the United States, 2011–2014

**DOI:** 10.5888/pcd12.140376

**Published:** 2015-02-19

**Authors:** Emily Bourcier, Diana Charbonneau, Carol Cahill, Andrew L. Dannenberg

**Affiliations:** Author Affiliations: Diana Charbonneau, Carol Cahill, Center for Community Health and Evaluation, Group Health Research Institute, Seattle, Washington; Andrew L. Dannenberg, University of Washington School of Public Health, Seattle, Washington.

## Abstract

**Introduction:**

The Center for Community Health and Evaluation conducted a 3-year evaluation to assess results of health impact assessments (HIAs) in the United States and to identify elements critical for their success.

**Methods:**

The study used a retrospective, mixed-methods comparative case study design, including a literature review; site visits; interviews with investigators, stakeholders, and decision makers for 23 HIAs in 16 states that were completed from 2005 through 2013; and a Web-based survey of 144 HIA practitioners.

**Results:**

Analysis of interviews with decision makers suggests HIAs can directly influence decisions in nonhealth-related sectors. HIAs may also influence changes beyond the decision target, build consensus and relationships among decision makers and their constituents, and give community members a stronger voice in decisions that affect them. Factors that may increase HIA success include care in choosing a project or policy to be examined’ selecting an appropriate team to conduct the HIA; engaging stakeholders and decision makers throughout the process; crafting clear, actionable recommendations; delivering timely, compelling messages to appropriate audiences; and using multiple dissemination methods. Challenges to successful HIAs include underestimating the level of effort required, political changes during the conduct of the HIA, accessing relevant local data, engaging vulnerable populations, and following up on recommendations.

**Conclusion:**

Results of this study suggest HIAs are a useful tool to promote public health because they can influence decisions in nonhealth-related sectors, strengthen cross-sector collaborations, and raise awareness of health issues among decision makers.

## Introduction

A health impact assessment (HIA) is used to communicate between public health professionals and decision makers in other sectors (eg, public policymakers) to increase stakeholder input and use of public health data in decisions that affect the public but are unrelated (or seemingly unrelated) to public health. HIAs convey to decision makers the potential health effects of proposed projects and policies such as those related to land use and transportation, and they make recommendations that promote the beneficial and mitigate the adverse health effects of such projects and policies. An HIA is “a systematic process that uses an array of data sources and analytic methods and considers input from stakeholders to determine the potential effects of a proposed policy, plan, program, or project on the health of a population and the distribution of those effects within the population. HIA provides recommendations on monitoring and managing those effects” (1).

Use of HIAs has been increasing since their introduction in Europe in the 1990s. Over 300 HIAs were completed or in progress in the United States as of mid-2014 (2). Prior reports called for evaluation to determine whether HIAs have the expected effects, to improve HIA methods, and to justify continuing investing in HIAs (1,3). Types of HIA evaluation include *process*, *impact*, and *outcome* evaluations (1). *Process* evaluation compares the processes followed in conducting the HIA with the investigators’ intended plans or the guidelines for an HIA (4,5). *Impact* evaluation, the focus of this article, examines the HIA’s effect on subsequent decisions and related events. *Outcome* evaluation examines changes in health status or health determinants resulting from the HIA; such evaluation is difficult and rarely done.

Two process evaluations (1 of 81 HIAs [6] and 1 of 23 HIAs [7]) in the United States show substantial variation in the conduct of HIAs in relation to practice guidelines and standards. Results of process evaluations were published for some individual HIAs (8,9). An impact evaluation of 88 HIAs, primarily in Europe, identified enablers and barriers to HIAs’ influencing decisions similar to those reported in this study (10). A process and impact evaluation of 17 case studies found that a wide variety of processes were being used by HIA practitioners in Europe (11). A 4-cell matrix for assessing HIA effectiveness developed in that evaluation was used in subsequent HIA evaluations (6,12). A process evaluation of 55 HIAs in Australia and New Zealand found that 65% of the reports that used a standard report review package were adequate (12). An impact evaluation of 11 of those 55 HIAs found most were effective, usually by directly influencing the decision process of nonhealth-related policymakers or raising stakeholder awareness of health issues (13).

The Robert Wood Johnson Foundation (RWJF), a major funder of HIAs in the United States, commissioned the Center for Community Health and Evaluation to study what constitutes success for HIAs, document their benefits, clarify and explain factors that contribute to successful HIAs, and look for opportunities to improve the HIA field. This is the first national study of HIAs that emphasizes the perspectives of decision makers, who are in the best position to indicate whether an HIA influenced their decision.

## Methods

We conducted a literature review of previous HIA evaluations and of factors that increase the likelihood of HIA success. HIA success was initially defined as the degree to which its recommendations were incorporated into a decision in a nonhealth related sector. A logic model (Figure) was created to define the intervention being evaluated, depict the intermediate and long-term outcomes that HIAs are intended to achieve, and guide development of the data collection instruments. An 8-member advisory committee with representatives from universities, philanthropic organizations, and HIA-focused organizations provided guidance on study design, interview and web-based survey questions, and analysis.

**Figure 1:**
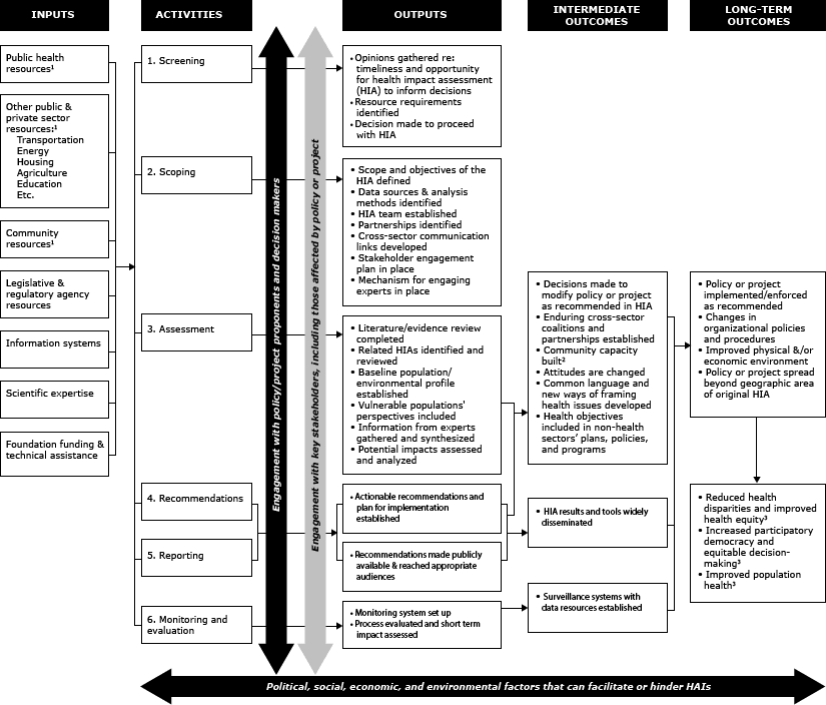
Logic model of a typical health impact assessment (HIA). The evaluators developed the logic model to depict the intermediate and long-term outcomes HIAs are intended to achieve and to guide development of the data collection instruments. The activities are aligned with the recommended six-step HIA framework (1).^ 1^ Includes financial resources, staffing, technical assistance, local knowledge, and advocacy. ^2^ Improved capacity to conduct HIAs, enhanced decision-making ability. ^3^ Outcomes that are not within the scope of this evaluation.

**Table:** 

Logic model of a typical health impact assessment (HIA). The evaluators developed the logic model to depict the intermediate and long-term outcomes that HIAs are designed to achieve and guide development of data collection instruments. The activities are aligned with the recommended 6-step HIA framework (footnote 1). The logic model is divided into 5 vertical sections labeled Inputs, Activities, Outputs, Intermediate Outcomes, and Long-Term Outcomes. The sections flow in a stepwise fashion from left to the right, starting with the first section, which lists the resources needed to conduct an HIA; an arrow to the next section lists the specific activities the HIA team will carry out with an arrow to the outputs that would occur as a result of the activities. Finally, arrows to the last 2 sections describe the changes one might see as a result — the outcomes. Each section has a box below each of the 5 headers as follows:
Inputs: Boxes beneath the header are labeled public health resources; other public and private sector resources (eg, transportation, energy, housing, agriculture, education); community resources (footnote 1); legislative and regulatory resources; information systems; scientific expertise; foundation funding and technical assistance.
Activities: Boxes beneath the header are labeled 1. Screening, 2. Scoping, 3. Assessment, 4. Recommendations, 5. Reporting, 6. Monitoring and Evaluation.
Outputs: Under the Outputs header, boxes of text define each of the 6 boxes under Activities as follows: Screening: Opinions gathered regarding timeliness and opportunity for an HIA to inform decisions; resource requirements identified; decision made to proceed with an HIA. Scoping: Scope and objectives of the HIA defined; data sources and analysis methods identified; HIA team established; partnerships identified; cross-sector communication links developed; stakeholder engagement plan in place; mechanism for engaging experts in place. Assessment: Literature and evidence review completed; related HIAs identified and reviewed; baseline population and environmental profiles established; vulnerable populations’ perspectives included; information from experts gathered and synthesized; potential impacts assessed and analyzed. Recommendations and Reporting: Actionable recommendations and plan for implementation established; recommendations made publicly available and reached appropriate audiences. Monitoring and evaluation: Monitoring system set up; process evaluated and short-term impact assessed,
Intermediate outcomes: Under the Intermediate Outcomes header, boxes of text are as follows: Decisions made to modify policy or project as recommended in the HIA. Enduring cross-sector coalitions and partnerships established; community capacity built (footnote 2). Attitudes changed. Common language and new ways of framing health issues developed. Health objectives included in nonhealth sectors’ plans, policies, and programs. HIA results and tools widely disseminated. Surveillance systems with data resources established.
Long-Term Outcomes:
Two boxes under the Long-Term Outcomes heading are connected with a vertical arrow.
Box 1: Policy or project implemented or enforced as recommended. Organizational policies and procedures changed. Improved physical and/or economic environment. Policy or project spread beyond geographic area of original HIA.
Box 2: Reduced health disparities and improved health equity. Increased participatory democracy and equitable decision making. Improved population health (footnote 3).
Two 2-way vertical arrows running from top to bottom of the model are labeled “Engagement with policy and project proponents and decision makers” and “Engagement with key stakeholders, including those affected by policy or project.”
A 2-way horizontal arrow running across the bottom of the model from the Activities column to the Long-Term Outcomes column is labeled “Political, social, economic, and environmental factors that can facilitate or hinder HIAs.” Includes financial resources, staffing, technical assistance, local knowledge, and advocacy. Improved capacity to conduct HIAs, enhanced decision-making ability. Outcomes that are not within the scope of this evaluation.

### Case studies

For this evaluation, the researchers used a retrospective comparative case study approach (14), including in-depth case studies of 23 completed HIAs. At the request of the evaluation’s funder, the study sample included 10 HIAs supported by the Health Impact Project, a collaboration of RWJF and The Pew Charitable Trusts, and 4 by the RWJF Active Living Research initiative. An additional 13 HIAs were found through consultation with HIA experts and considered by them to have elements that were successful. The study team recruited the investigators of 23 of these 27 HIA cases to participate in the evaluation; 2 HIA investigators declined to participate, 1 HIA did not self-identify as an HIA, and 1 HIA was replaced by another to add more geographic and investigator diversity. Overall the sample was purposefully selected for variety in geography, sector, and funding source.

The investigators scheduled site visits at least 6 months after the HIA was released for 21 cases; less time had passed for the other 2 cases. The evaluation team worked with each HIA lead investigator to identify key informants and then scheduled interviews with the HIA team, 1 or 2 decision makers, and 1 or 2 community stakeholders for each HIA.

The investigators developed a semi-structured interview guide, with open-ended questions tailored to HIA team members, decision makers, and community stakeholders. Questions covered origins of the HIA, decision maker and stakeholder involvement, time and resource use, how the HIA used data to support its recommendations, the degree to which recommendations were implemented, major impacts of the HIA, factors that facilitated successes or created challenges, and opportunities to increase the success of HIA. Detailed narrative data were gathered on each HIA from multiple perspectives. Decision makers included federal, state, and local elected and appointed officials; high-level agency staff; and private sector leaders. Interviews ranged from 30 to 90 minutes and were conducted between March 2012 and March 2013 with 166 key informants: 119 interviews were in person and 47 were by phone. Informants were 78 HIA practitioners, 47 decision makers, and 41 community members. For quality assurance, 6 of the 23 HIA site visits were conducted by 2 members of the evaluation team, and interviews for 2 HIAs were independently coded by 2 people.

Transcripts were uploaded into the qualitative analysis software program Atlas.ti 6.0 (Atlas.ti, Corvallis, Oregon). Using grounded theory techniques (15) and a code list based on the evaluation questions and the logic model, team members who conducted the interviews coded the data. The coded data were analyzed using an immersion/crystallization approach (16), which emphasizes gaining an in-depth knowledge of the data to identify key themes. Themes were aggregated into a case study template. Each case study was reviewed by all 3 team analysts to ensure accurate representation of the data. The evaluation team then reviewed the data again to identify cross-cutting themes and draw conclusions. The team organized the evaluation domains of interest (eg, impact, success factors, challenges) into 8 tables to synthesize the themes across the 23 cases (17).

### Web-based survey

In January 2013, a national Web-based survey of HIA practitioners was sent via SurveyMonkey (www.surveymonkey.com) to a convenience sample of 121 people associated with HIAs who were identified from the Health Impact Project and University of California, Los Angeles–HIA websites. In March 2013 this survey was sent to an additional 22 people who were among attendees of the HIA of the Americas meeting. In February and March 2013 the survey was promoted to recipients of email newsletters from the Health Impact Project and Human Impact Partners. Multiple reminders were sent, and the survey closed in April 2013. The questions were primarily closed-ended and focused on the impact of HIAs and success factors.

## Results

### Case studies

The sample consisted of 23 HIAs in 16 states completed from 2005 through 2013 (Table 1). Sectors represented among these HIAs were 11 built environment, 3 transportation, 3 natural resources and energy, 2 food and agriculture, 1 housing, 1 economic policy, 1 climate change, and 1 labor and employment. Of the 23 HIAs, 14 were funded by RWJF, 4 by government agencies, 3 by other foundations, and 2 by other sources.

**Table 1:** Characteristics of 23 Health Impact Assessments (HIAs) Examined in Depth in Center for Community Health and Evaluation HIA Evaluation Study, 2011–2014

HIA	Year Completed	Lead Agency	Sector	Focus	Primary Funding Source	State
1	2013	University	Transportation	Mass transit expansion	Foundation	California
2	2012	Nonprofit organization	Food and agriculture	Agricultural plan implementation	Foundation	Hawaii
3	2012	Nonprofit organization	Natural resources and energy	Metering technology implementation	Foundation	Illinois
4	2012	University	Built environment	Transit-oriented development	Foundation	Texas
5	2011	University	Built environment	Brownfield site redevelopment	Foundation	Georgia
6	2011	Nonprofit organization	Transportation	Light rail expansion	Foundation	Minnesota
7	2011	State policy agency	Economic policy	State budget	Foundation	New Hampshire
8	2011	Nonprofit organization	Food and agriculture	Farm-to-school legislation	Foundation	Oregon
9	2011	Local health department	Built environment	Waste recycling facility permitting	Foundation	New Mexico
10	2011	State health agency	Labor and employment	Worksite tax-credit legislation	Foundation	Kentucky
11	2010	State health agency	Climate change	Cap-and-trade regulations	Foundation	California
12	2010	University	Built environment	Zoning code revision	Foundation	Maryland
13	2010	University	Built environment	Urban revitalization	Foundation	Missouri
14	2010	Local health department	Built environment	Bicycle-pedestrian master plan	Foundation	Washington
15	2010	Local health department	Built environment	Alcohol outlet regulation	Federal agency	California
16	2010	Local health department	Built environment	Comprehensive plan update	Federal agency	Oregon
17	2010	University	Natural resources and energy	Oil and gas exploration	Local agency	Colorado
18	2010	University	Transportation	Pedestrian infrastructure development	Federal agency	New Mexico
19	2008	Local health department	Built environment	Comprehensive plan update	Foundation	California
20	2008	Nonprofit organization	Built environment	Transit-oriented development	Foundation	California
21	2007	University	Built environment	Urban redevelopment	Foundation	Georgia
22	2007	Local agency	Natural resources and energy	Environmental Impact Statement mitigations for oil and gas project	University	Alaska
23	2005	University	Housing	State rental voucher budget	Anonymous donor	Massachusetts

### Identified impact

Table 2 presents the identified impacts of the 23 HIAs. In 11 HIAs, decision makers reported their decisions would have been markedly different without the HIA. Decision target outcomes — the way projects, plans, and policies were developed or implemented — could be directly linked to specific HIA recommendations in 11 HIAs. This, in turn, altered the trajectory of a policy or plan in ways that were intended to improve health or mitigate potentially adverse health consequences. Fourteen HIAs influenced changes beyond the decision under consideration, resulting in the incorporation of health objectives into plans, policies, and programs established by nonhealth-related agencies, and 8 HIAs — some of which overlap with the aforementioned 14 — contributed to longer-term outcomes beyond initial decision targets.

**Table 2:** Reported Impacts of Health Impact Assessments (HIAs) in Case Study Sample (N = 23), With Illustrative Quotes and Examples, Community Health and Evaluation HIA Evaluation Study, 2011–2014

Impact on Decision Making	No. of HIAs (N = 23)	Illustrative Quote or Example
**Direct impacts**		
Decision maker reported HIA shaped their decision making	11	“Recommendations helped shape how we proceeded about land acquisition and project prioritization . . . and over the last couple years we kicked off a health initiative where we connect the built environment to the health of community and HIA recommendations have been huge in that.” — Local decision maker
Direct and concrete contributions from the recommendations to the decision-making process	11	A state decision maker indicated that the HIA shaped the direction of his decision to include urban forests in carbon emissions plans.
Incorporation of health objectives into plans, policies, and programs of nonhealth-related agencies	14	A federal decision maker reported that after the HIA he now raises the issue of health analysis with every plan he is involved in at his nonhealth-related agency. “I raise this every time I’m on the periphery of another plan. I ask, ‘Are you going to do a public health analysis? Is this an issue? If so, you should do an analysis.’”
Longer-term outcomes beyond initial decision targets	8	One federal agency is now incorporating public health considerations in environmental impact statements.
**Other impacts**		
Institutionalized or strengthened existing relationships between individuals and organizations, or created new and enduring relationships between public health and other agencies such as transportation or planning departments	17	One HIA resulted in a shared staff position between county public health and planning departments.
Helped decision makers and stakeholders see how health is connected to seemingly unconnected issues	16	“It made policymakers like myself understand that you pay one way or another.” – Former state representative
Built consensus around controversial topics	9	“Before there was hatred, now there is tolerance.” — HIA practitioner
Amplified community member voices in the decision-making process	9	“Now when I go talk at a DOT or local transportation meeting, I can say, ‘According to the HIA . . .’ rather than just being an angry mom.” — Community stakeholder

Abbreviations: DOT, Department of Transportation.

HIAs contributed to various other impacts. Seventeen HIAs institutionalized or strengthened pre-existing relationships among individuals and organizations, or they created new and enduring relationships between public health and other agencies such as transportation or planning departments. Sixteen HIAs helped decision makers and stakeholders understand how health is connected to seemingly unconnected issues. HIAs helped build consensus around controversial topics. In 9 HIAs, the process amplified community member voices in the decision making process.

### Reported success factors

The evaluation identified components that HIA teams can incorporate into their assessments to increase the likelihood of HIA success. These components (or “success factors”) clustered into 7 themes (Table 3):

**Table 3:** Reported Success Factors and Challenges to Conducting Health Impact Assessments (HIAs), With Illustrative Quotes and Examples, Community Health and Evaluation HIA Evaluation Study, 2011–2014

HIAs (N = 23)	Number of HIAs	Illustrative Quote or Example
**Success factor**		
Screen and choose HIA targets wisely — an HIA is not always the right tool	19	The HIA was initiated at the start of a private developer’s planning process and as they were conducting a survey and community engagement; the HIA team was able to participate in both.
Invest in the right team to conduct HIA	15	An HIA team needs people with competence in at least 3 key roles: “. . . someone who coordinates facilitation, someone to coordinate the data (ideally, with HIA expertise), and a content specialist.” — HIA practitioner
Engage key stakeholders	14	“There was a constituency [people paying attention and advocating for alcohol outlet changes] advocating — that’s why we kept the liquor density piece in.” — Local decision maker
Engage decision makers throughout the process	10	A planner decision maker’s support and guidance made it easier to incorporate the HIA into a county comprehensive plan update — “[the HIA was] like connective tissue.”
Craft clear recommendations that spark action	9	“It is critically important to craft recommendations that are actionable by the decision maker, rather than simply writing recommendations that make sense from a public health perspective but which there may be no legal way for a policymaker to implement.” — HIA practitioner
Deliver compelling messages to the right audiences at the right times	6	“Say it 10 times, in 10 different ways.” – HIA practitioner. “I think for practitioners of HIA getting the word out in ways that the public and the public’s representatives can understand — so not just technical jargon — is important . . . it needs to be translated.” — State decision maker
Take advantage of HIA credibility	11	[HIA] was “the only planning document that has research behind it.” — County administrator
**Challenge**		
Underestimating overall level of effort	19	“We provided in-kind resources — staff time. Probably doubled or tripled the grant amount with this in-kind.” – HIA team member. “Having a dedicated staff person is important — [it is] difficult to fit HIA work on top of regular work.” — HIA team member
Engaging stakeholders and decision makers	18	Competing community priorities present challenges for engagement and implementation of HIA recommendations — the county commissioners wanted to make things easy for new development, whereas developers wanted to keep costs low and not put in health-promoting infrastructure unless taxpayers pay for it; citizens wanted to slow traffic, leading to narrower road standards, whereas the fire department wanted wider streets for their trucks.
Pace of decision making and political administration changes	8	The community steering committee leading the HIA was unable to keep up with and respond to the fast pace of the political process.
Access to relevant data	8	Statistics about potential impact on those in the neighborhood, which the recycling plant developers included in their application documentation, were moving targets that were difficult to obtain.
Consistently and meaningfully incorporating equity and vulnerable populations	23	Experiences were inconsistent, ranging from no experience at all (especially in areas that were not culturally or economically diverse), to including disparities data in the HIA report, to engaging stakeholders who could articulate concerns of vulnerable populations.
Following up on recommendations	10	“The message I would want to give to public health professionals is after they complete their work (the HIA) it’s not enough to put it out to the medical community, but to really try and get in touch with the people who can look over the study and apply its findings in practical ways.” — State official

**Screen and choose HIA targets wisely** because different decision contexts require different HIA approaches, and HIA is not always the right tool. To help decide whether to conduct an HIA and to determine its scope, HIA practitioners recommend the HIA screening step include assessment of the following issues: whether momentum is already building for the issue, whether the decision makers have basic knowledge about health-related issues, what relationships exist between those conducting the HIA and the decision makers, and how the HIA timing aligns with the decision-making process.

**Invest in the right team to conduct the HIA**. The composition of the HIA team is crucial, since successful HIAs rely on many kinds of expertise and require sustained collaborative effort. Teams should include experts in the content related to the decision under consideration, knowledge of the decision-making process, and skills in project management and stakeholder engagement.

**Engage key stakeholders.** Community members, influential champions, and other stakeholders can build momentum for considering and adopting HIA recommendations. Stakeholders are valuable sources of political expertise and community knowledge.

**Engage decision makers throughout the process.** Bringing decision makers on board as stakeholders or team members increases the likelihood that HIA recommendations will receive appropriate consideration.

**Craft clear recommendations that spark action.** To increase the likelihood of adoption and implementation, recommendations should be actionable, realistic, and sector-specific; consider the implementer’s authority to act; and address timelines and costs. A decision maker reading an HIA report should have a clear idea of what to do next.

**Deliver compelling messages to the right audiences at the right times.** HIA teams should make complex information accessible to many audiences throughout the HIA process. In retrospect, some HIA team members wished they had communicated HIA information through multiple channels and earlier, not only at the recommendations stage. Many advised that HIAs should consider both the content and strategic timing of their dissemination efforts and tailor these to the needs of different audiences (eg, decision makers, business audiences, community members).

**Take advantage of HIA credibility.** Because HIAs combine stakeholder input and robust public health evidence, they can provide a credible evidence-based rationale for recommendations in the setting of complex policy and planning decisions.

### Reported challenges

Common challenges to conducting successful HIAs cluster into 6 themes (Table 3). For most HIAs, the time and resources required to conduct the HIA were greater than expected. Engaging stakeholders and decision makers proved more difficult than anticipated for most HIAs because of competing time demands and political considerations. In some cases the decision-making process moved faster than the HIA process, thereby undermining the usefulness of recommendations.

In some cases, relevant data were not available at a sufficiently local level to document disparities in impacts. Some HIA investigators reported difficulties in engaging members of vulnerable populations, and approaches used reflected different purposes and philosophies. Finally, adequate dissemination and follow-up on implementation were absent for many HIAs, thus reducing the likelihood of the recommendations having an impact on decisions.

### Web survey results

Of the 173 survey respondents, 144 had conducted at least 1 HIA and self-identified as HIA practitioners. In this convenience sample, the number of practitioners who received the survey and the resulting response rate are unknown. Considering the number of HIAs completed in the United States, the survey respondents probably constitute a substantial proportion of all US HIA practitioners. Of the 144 respondents, 47% worked for government agencies, 23% for academic institutions, 20% for nonprofit organizations, 7% for for-profit organizations, and 2% for other entities. The 135 respondents who identified their location were from 1 of 32 states or 5 non-US countries. The amount of overlap between the anonymous survey respondents and case study informants is unknown.

In response to a closed-ended question, the most important outcomes expected by respondents from an HIA were recommendations’ influence on decision-making (87%), inclusion of health-related objectives in other sector plans (68%), enduring cross-sector partnerships (64%), a common language and new ways of framing health issues (35%), increased community capacity to make decisions and conduct HIAs (33%), and changed attitudes (25%). For respondents’ most successful HIA completed to date, the response of the decision maker to the HIA was reported as “thrilled” for 8% and “receptive” for 62%; 15% of respondents reported a mixed decision maker response, 10% a neutral response, 3% decision maker pushback, and 2% did not know. Eighty percent reported engaging representatives from vulnerable populations in their most recent HIA.

Most respondents (84%) had been involved in an HIA they considered successful. Types of success observed by these respondents included contributions to cross-sector coalitions or partnerships (35%), influence on decision-making (19%), and contributions to positive, detectable changes in the community (6%). These proportions of observed impacts are lower than the respondents’ expected impacts noted above.

## Discussion

By obtaining information directly from decision makers who know what influenced their decisions, this impact evaluation provides strong evidence that HIAs can influence decisions to promote health considerations in projects and policies outside of the health sector. This study also documents how HIAs can influence changes beyond the decision target, by raising decision makers’ awareness of the effects of their decisions on people’s health and by giving community members a stronger voice in decisions that affect them. Success for HIAs should therefore be defined by both their impacts on decisions as well as on the environments in which those decisions are made.

On the basis of study results, we have several recommendations for improving the conduct of HIAs and building the field. Pay more attention to the needs of vulnerable populations by focusing on practical factors such as the resources and skills needed, the HIA timeline, and how shared expectations are developed and managed. HIA teams could make strong calls for action to increase the likelihood of the recommended actions being implemented. Provide an implementation plan in their recommendations, including a list of who should be responsible for each task.

Some HIAs are done by investigators with little HIA experience who welcome technical assistance. We have recommendations also for providers of such technical assistance: focus more on HIA components that are challenging, such as engaging stakeholders in meaningful ways (18,19); cultivate relationships with decision makers and potential adversaries; facilitate high-functioning HIA teams; obtain and use locally relevant data; tailor separate communication products for various audiences; and suggest tools to help prospective HIA investigators determine whether an HIA is appropriate and how to define its scope.

On the basis of information from key informants, we have several recommendations for funders on how to improve the success of HIAs. For example, be more flexible about timelines and deliverables because HIA teams must often respond to changing political and decision-making conditions; build community stakeholder engagement into their rating criteria, and fund elements that support community engagement, such as facilitation training and food for meetings; and require and support an implementation plan as part of the HIA report, while recognizing that the plan may extend beyond the original funding period.

This report adds to the growing literature on the evaluation of HIAs as a field (6–13). Study strengths include the decision maker perspective on impact and which elements make HIA more influential and useful, its relatively large and diverse sample size, and the resources to gather data from multiple perspectives. Study staff achieved excellent cooperation with investigators, stakeholders, and decision makers of the individual HIAs and conducted over 120 hours of in-person interviews with these key informants. The consensus-based approach and use of 3 evaluation staff members to both gather and analyze the data ensured rigorous analysis of the enormous amount of qualitative data. This study was also strengthened by the expertise of its investigators in evaluation and in HIA, by interactions with an experienced advisory committee, and by collaboration with investigators of other HIA evaluations simultaneously under way in the United States and Australia.

Several limitations should be considered in interpreting study results. First, the 23 HIAs examined in this report are not necessarily representative of all HIAs in the United States; the sample was purposefully selected for diversity and was not a random sample. Second, it is difficult to separate impacts of HIA from other influences on decisions. In any decision, health considerations need to be balanced with economic, political, social, and other factors, and a decision maker may be unable to state how much weight was given to each factor. Third, the amount of time from the HIA’s completion to the informant interviews varied. In some cases decisions had been made several years ago, thus introducing possible recall bias, whereas in other cases decisions were ongoing at the time of the interview. Fourth, some HIAs involved politically sensitive topics about which some informants may have been reluctant to provide full information.

This report documents that HIAs can influence decision making and strengthen cross-sector collaborations in ways that improve the health of a community. The most promising tactics for increasing HIA effectiveness include choosing HIA targets well, gathering the right HIA team, engaging stakeholders and decision makers, creating actionable recommendations, delivering compelling messages, and establishing and following through on an implementation plan.
